# Effects of Tibetan Singing Bowl Intervention on Psychological and Physiological Health in Adults: A Systematic Review

**DOI:** 10.3390/healthcare13162002

**Published:** 2025-08-14

**Authors:** Fei-Wen Lin, Ya-Hui Yang, Jiun-Yi Wang

**Affiliations:** 1 Department of Healthcare Administration, Asia University, Taichung 413305, Taiwan; 2 Wuchi District Public Health Center, Taichung 435051, Taiwan; 3 Department of Medical Research, China Medical University Hospital, China Medical University, Taichung 404333, Taiwan

**Keywords:** Tibetan Singing Bowl, anxiety, depressive symptoms, heart rate variability, brainwave activity

## Abstract

**Background**: Anxiety and stress are common mental health issues that affect both psychological and physiological well-being as well as quality of life. The Tibetan Singing Bowl, which combines sound and vibration, is often used in meditation and relaxation and may offer therapeutic benefits. However, current research findings are scattered and lack systematic integration and quantitative validation. **Methods**: This study is a systematic review that included 14 quantitative studies from the past 16 years investigating the effects of Tibetan Singing Bowl interventions on adult psychological and physiological health. Data were sourced from six major databases and supplemented through citation tracking. Inclusion criteria were adults aged 18 and over, with interventions primarily involving Tibetan Singing Bowls, and reporting quantitative outcomes related to psychological indicators (e.g., anxiety and depressive symptoms) and physiological indicators (e.g., Heart Rate Variability and brainwave activity). Study quality was assessed using Joanna Briggs Institute (JBI) criteria, and findings were synthesized narratively to identify patterns and trends. **Results**: Study populations included general adults, individuals with emotional distress, and patients with cancer or chronic illnesses. Interventions ranged from single sessions to multiple courses, with some incorporating breathing or other practices. Most studies reported significant reductions in anxiety and depressive symptoms, improvements in well-being and quality of life, increases in Heart Rate Variability, and decreases in heart rate. Some studies also found increased Delta and Theta brainwave activity. Due to heterogeneity in study design and limited articles, no meta-analysis was conducted. **Conclusions**: Tibetan Singing Bowl interventions demonstrate potential for stress reduction and psychological well-being, offering a non-invasive, low-risk, and widely accepted complementary method supporting therapeutic processes, which can be suitable for clinical and community settings. Future research should focus on rigorously designed controlled trials and consider follow-up assessments to more accurately evaluate the effectiveness of TSB interventions.

## 1. Introduction

Anxiety and stress have become critical global public health concerns, closely associated with various chronic conditions, including cardiovascular disease, hypertension, diabetes, and all-cause mortality [1,2]. According to a systematic review, the global prevalence of anxiety disorders ranges from 3.8% to 25% [3]. These psychological issues pose not only a threat to individual health but also long-term burdens on healthcare systems and society, making them a key focus within non-communicable disease (NCD) policy agendas [4].

Although medication and psychotherapy are commonly used interventions, their real-world application faces several challenges, including side effects, limited accessibility, and low treatment adherence [5,6,7]. As a result, increasing attention has been directed toward feasible, non-invasive, and drug-free alternative interventions. Among these, sound therapy has garnered growing interest due to its low risk and high acceptability in both academic and clinical settings [8,9,10].

The Tibetan Singing Bowl (TSB) and associated instruments originate from the Himalayan regions of Nepal, Tibet, and India, where they have traditionally been used in religious rituals, meditation, and spiritual healing practices [9]. These bowl-shaped instruments, cast from metal alloys, produce rich overtones and sustained resonance, embodying both sonic and vibrational properties, and have commonly been used in shamanic ceremonies and monastic contemplative practices [11]. Nowadays, TSBs have crossed cultural boundaries and have been widely applied in sound therapy, stress reduction, mindfulness practices, and psychological relaxation [10,11].

TSB intervention, like other forms of music therapy, shares similar clinical applications and underlying mechanisms. Although the precise mechanisms behind its potential therapeutic effects are not yet fully understood, several plausible explanations have been proposed. First, the neurotransmitter dopamine associated with music-induced pleasure plays a key role in neuroplasticity and behavioral learning [12,13]. Second, music therapy has been shown to reduce cortisol levels, thereby alleviating stress. One proposed mechanism of music therapy involves modulation of the cortico-hypothalamic speech circuit, with music exerting a regulatory effect on the amygdala, leading to reduced emotional reactivity [12,14,15]. Third, music may influence brain wave patterns, especially when individuals listen to calming music or the resonant tones of singing bowls. When singing bowls are applied directly to various parts of the body, they can produce vibrations that stimulate brainwave activity and promote whole-body resonance [16].

The TSB, as a complementary and alternative medicine tool that integrates sound and vibration, has been recognized for its potential to regulate emotions and promote nervous system relaxation [17,18]. Some studies suggest that TSB sound interventions may reduce heart rate, enhance heart rate variability (HRV), and modulate brainwave activity by decreasing beta waves and enhancing theta waves, thereby facilitating deep relaxation and mindfulness [18,19,20]. However, existing studies vary widely in sample sizes, intervention designs, and assessment indicators, which makes it difficult to integrate and compare findings [21].

To date, only two review articles have examined the intervention effects of TSB. One systematic review [22] included only four studies, consisting of two randomized trials and two single-group intervention studies. Moreover, it lacked a quality assessment, making it difficult to assess the certainty of evidence and draw definitive conclusions. The other narrative review [21] included 27 articles that also comprise the history and cultural significance of TSB. Although the review article offered a concise overview of current evidence on TSB’s health benefits, it lacked a systematic approach to literature review, did not conduct quality assessment for individual studies, and failed to summarize key findings by specific outcome variables. Therefore, there is significant value in conducting a rigorous and comprehensive systematic review of TSB interventions.

The present study aims to conduct a systematic review of quantitative studies on the effects of TSB sound intervention on psychological outcomes (e.g., anxiety, depressive symptoms, and quality of life) and physiological outcomes (e.g., HRV and brainwave activity) in adults. It also explores the potential impact of various intervention conditions (e.g., frequency, duration, and intervention settings) on outcomes, while evaluating the methodological quality and limitations of the existing literature. This review seeks to bridge existing knowledge gaps and provide an evidence-based foundation for future practical applications and research design.

## 2. Methods

This systematic review was registered on Protocols.io (DOI: 10.17504/protocols.io.j8nlkdbmxg5r/v1; protocol ID: 124701), and the study selection process followed the PRISMA 2020 guidelines. A comprehensive literature search was conducted to identify all published studies related to TSB interventions.

### 2.1. Search Strategy

A literature search was conducted across six electronic databases: MEDLINE, PubMed, Scopus, EMBASE, CINAHL, and Cochrane. Boolean operators were applied using keywords including “Singing Bowl” OR “Tibetan Bowl” (see the search strings in Supplementary Table S1). The search was restricted to peer-reviewed journal articles published between 2000 and March 2025, aiming to ensure contemporary relevance and exclude outdated literature. To broaden the scope of the search and identify relevant articles that may have been missed, manual citation tracking was performed by reviewing the reference lists of included studies. The initial search was completed in April 2025.

### 2.2. Study Selection Criteria

This systematic review aimed to identify original quantitative empirical studies investigating the effects of TSB interventions on psychological or physiological outcomes. Given the limited number of existing studies on TSB interventions, this review aimed to provide a comprehensive overview of the current evidence base and research trends. To capture the breadth of available data, we included both randomized controlled trials (RCTs) and quasi-experimental studies (QESs). The latter encompassed non-randomized controlled studies as well as single-group pre-post designs. While these QESs are methodologically weaker, they offer preliminary insights into potential effects and inform future research directions. No specific outcome variables were predetermined prior to the systematic search, i.e., any study examining psychological or physiological changes was considered. The target population was adults, and participants were required to have received a TSB intervention. Due to feasibility, resource limitations, and time constraints, only studies with titles and abstracts available in English were included.

Exclusion criteria included non-original studies (e.g., literature reviews, conference abstracts, commentaries, case reports), studies combining TSBs with other sound-based treatments (e.g., incorporating gongs, background music, or other musical instruments in sound baths) due to the involvement of multiple sound sources, studies lacking complete data, and studies not published in peer-reviewed journals (e.g., theses, book chapters, technical reports, grey literature).

In this review, a TSB intervention was defined as any treatment session that involved the use of TSBs played manually to produce sound and vibration. Studies were included regardless of whether the bowls were placed on the body or used in a non-contact setting. Both individual and group sessions were eligible. Additionally, studies relying on pre-recorded audio playback without live bowl use were eligible.

### 2.3. Data Extraction and Management

The researchers developed a standardized data extraction form with predefined fields to ensure consistency and clarity in data structure. Two authors (FWL, YHY) independently screened the titles and abstracts to determine eligibility based on the inclusion criteria. Disagreements were resolved through discussion, and, if necessary, a third reviewer served as an adjudicator. Before formal screening, a pilot screening was conducted, including both eligible and ineligible studies, to test the inter-rater consistency between the two reviewers. Data extraction was also conducted by the two authors independently. Extracted items included: author, year of publication, study location, study population, sample size, intervention and control groups, duration and frequency of the intervention, outcome measurement methods, and outcome data. All data were processed in accordance with the *Cochrane Handbook for Systematic Reviews of Interventions*.

### 2.4. Quality Assessment Tools

This study employed the Joanna Briggs Institute (JBI) critical appraisal tools. For randomized controlled trials (RCTs), the JBI Critical Appraisal Checklist for Randomized Controlled Trials was used [23], which includes 13 assessment items. For quasi-experimental studies (QES), the JBI Critical Appraisal Tool for Quasi-Experimental Studies was applied [24], consisting of nine evaluation criteria. All studies were independently assessed by two reviewers (FWL, YHY). Any disagreements were resolved through discussion or by consulting a third expert for consensus when necessary. Risk of bias results were presented graphically, with low risk indicated by a green “+”, unclear risk by a yellow “–”, and high risk by a red “×”. The certainty of evidence was further evaluated using the GRADE approach (Grading of Recommendations Assessment, Development and Evaluation). Meanwhile, a summary of findings was conducted for each outcome. The certainty rating was downgraded when the included studies exhibited a high risk of bias or serious concerns about certainty.

### 2.5. Data Analysis

To ensure inter-rater consistency in screening and quality appraisal, Cohen’s kappa coefficient was calculated. A kappa value of 0.41–0.60 suggests moderate agreement, 0.61–0.80 substantial agreement, and 0.81–1.00 almost perfect agreement. In addition, Cohen’s *d* was calculated as the effect size for each outcome variable when data were available. An effect size of 0.2 suggests a small but noticeable difference, 0.5 a moderate and meaningful difference, and 0.8 a large and substantial difference.

## 3. Results

### 3.1. Screening Results

**Figure 1:**
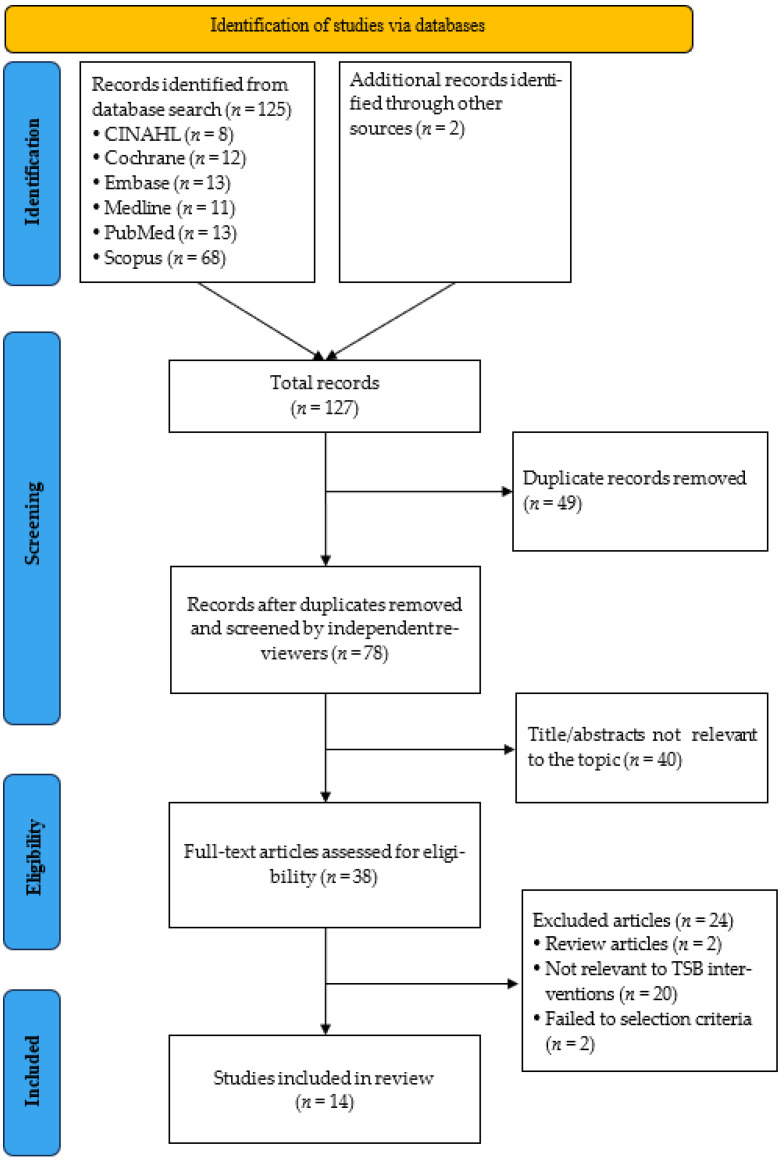
PRISMA flow diagram of study selection.

A total of 127 records were identified through the database search. After removing 49 duplicates, 78 articles remained for initial screening. Based on titles and abstracts, 40 articles that were irrelevant to the research topic were excluded, leaving 38 articles for full-text review. Ultimately, 14 studies met the inclusion criteria and were included in the systematic review (see PRISMA Flow Diagram in Figure 1; see the list of excluded articles and the reasons in Supplementary Table S2). Among them, two articles were derived from the same study and analyzed different outcome variables; therefore, they were considered as a single study and evaluated together [9,17]. During the screening and quality appraisal stages, excellent agreement was observed between the two reviewers, with Cohen’s kappa coefficients higher than 0.9.

The heterogeneity of study designs limits the feasibility of synthesis. Aside from single-group studies, an outcome variable could be assessed in only two or three RCTs or QES with control groups. Therefore, meta-analyses and related quantitative statistics were not performed in this review.

### 3.2. Characteristics of Studies

To distinguish the study design and its evidentiary level, the characteristics of the 14 included studies are summarized and presented in Table 1, Table 2 and Table 3 according to study design: RCTs, QES with control groups, and single-group pre-post designs. The publication years ranged from 2000 to 2025.

In terms of geographic distribution, two studies each were conducted in the United States [8,9], Russia [29,31], and Austria [11,27]. The remaining eight studies were conducted in the following countries, one study each: South Korea [20], Germany [18], Greece [26], the Netherlands [28], Italy [19], India [30], Iran [10], and Chile [25].

Regarding study design, the included studies comprised three parallel-group RCTs [25,26,27] and two crossover trials [8,11], four QESs with controls [10,28,29,30], and five single-group pre-post studies [9,18,19,20,31].

Sample sizes in individual studies ranged from 12 to 81 participants. Most studies involved healthy or general adult populations. However, several studies focused on specific groups, such as cancer patients [19], individuals with post-traumatic stress disorder [28], and cardiovascular patients [10]. One study targeted adults with chronic nonspecific spinal pain, aged from 20 to 60 years [27].

### 3.3. Intervention Methods, Duration, and Control Designs of Included Studies

The reviewed studies were categorized by intervention method into contact-based and non-contact-based approaches. Contact-based interventions involved placing TSB directly on the body during playing, such as on the palm [10], back [27], or pain site [19]. Some studies placed bowls on multiple body locations [18,29,31]. Non-contact-based interventions did not involve direct physical contact with the body. These included placing bowls around or beside the head [8,9,30], playing pre-recorded bowl sounds [20], suspending bowls beneath a hammock [11], combining bowls with color visualization and breathing exercises [28], and having participants sit quietly with eyes closed while listening to live improvised bowl performances [25,26].

Intervention duration and frequency varied across studies. Seven studies implemented single-session interventions [9,10,18,20,25,29,30], with session lengths ranging from 7 to 70 min. Six studies employed multiple-session interventions, comprising 2 to 12 sessions, with each session lasting from approximately 15 to 30 min [8,11,26,27,28,31]. Notably, only one study [19] conducted an intervention over three months, which consisted of six 60 min sessions.

Among the nine studies with control groups, the control conditions varied. These included: Progressive Muscle Relaxation (PMR), quiet resting, or no intervention [25]; a placebo condition using a silent bowl and standard care group [27]; pre-recorded relaxing music (MP3) and standard treatment [26]; psychological support and stress education [28]; routine medical care instructions [10]; sound-only exposure without physical contact [29]; silent sitting as a part of a crossover comparison [8]; lying quietly without striking the bowl [11]; and quiet resting without sound stimulation [30].

### 3.4. Measurement Tools

Anxiety assessment tools included the Spielberger–Khanin Anxiety Scale (SKAS) [29], the Hospital Anxiety and Depression Scale (HADS)—anxiety subscale [9,19], the State-Trait Anxiety Inventory (STAI) in its full form [31] or a subscale (State Anxiety Inventory, SAI) [10,25], and the Zung Self-Rating Anxiety Scale (ZSAS) [31]. Depressive symptoms assessment tools included the HADS—depression subscale [9,19], Zung Self-Rating Depression Scale [29], and Symptom Checklist-90-Revised—Depression Subscale [26]. Stress assessment tools included the Tension subscale of the Profile of Mood States—Short Form (POMS-SF) [9] and the Distress Thermometer (DT) [19].

Well-being was assessed using the Functional Assessment of Chronic Illness Therapy—Spiritual Well-being (FACIT-SP) [9], the psychological health-related subscales of the 36-Item Short Form Health Survey (SF-36), including vitality, emotional functioning, and social functioning [28], and the Well-being, Activity, and Mood Scale (WAM) [29]. Quality of life was assessed using the SF-36 [27], and its selected subscales, including vitality, social functioning, emotional functioning, and psychological health [28], and the vitality subscale alone [19].

These psychological instruments were all self-reported and, therefore, subjective in nature. Nonetheless, most demonstrated good reliability and validity according to previous validation studies. For example, the STAI, which was used in the included studies [10,25,31], has demonstrated excellent internal consistency (Cronbach’s α ≈ 0.86–0.95) and strong cross-cultural construct validity in prior research. Similarly, the HADS, used in studies [9,19], has shown acceptable internal consistency (α ≥ 0.80) and good discriminant validity. The SF-36, a well-established and widely used tool for assessing health-related quality of life and employed in studies [19,27,28], has demonstrated good reliability in validation research (α ≈ 0.70–0.94 in the psychological domain). Finally, instruments such as the ZSAS and SCL-90-R [9,26,31] have reported Cronbach’s α values ranging from 0.85 to 0.92 in previous literature. These findings suggest that the instruments selected were appropriate for detecting emotional distress and psychological changes, with demonstrated sensitivity to intervention effects.

Heart Rate Variability (HRV) was the most commonly used physiological indicator, encompassing time-domain measures (e.g., Root Mean Square of Successive Differences, RMSSD), frequency-domain measures (e.g., High Frequency, HF), and nonlinear parameters [18,19,25,27,29,30]. Heart Rate (HR) was also a commonly used measure [8,19,30,31]. Additionally, some studies assessed autonomic nervous system responses using Skin Conductance Level (SCL) [19].

Regarding neural activity, electroencephalography (EEG) was used to assess the effects of sound stimulation on brain rhythms. The analyzed brainwave frequency bands included Delta, Theta, Alpha, Beta, and Gamma waves [18,19,20,25]. Details of the study participants, intervention methods, and measurement tools for each study have been summarized according to study design in Table 1 (RCTs), Table 2 (QES), and Table 3 (pre-post single-group designs).

Heart rate variability (HRV) indices—including RMSSD, HF, and LF/HF—showed high intraclass correlation coefficients (ICC > 0.80), reflecting their sensitivity to stress-related autonomic changes. EEG recordings of Delta, Theta, and Alpha bands exhibited moderate to high test–retest reliability, supporting their use in relaxation and mindfulness studies.

### 3.5. Effects of the Interventions

#### 3.5.1. Psychological Indicator: Anxiety

Findings from two controlled trials investigating anxiety suggest that TSB interventions had significant effects on certain anxiety indicators. In both studies, scores on State Anxiety Inventory were significantly lower in the intervention group than in the control group (between-group difference: *p* < 0.001 [10,25]), with Cohen’s *d* = 1.51 and 2.18 (estimated according to the provided data).

In four single-group studies, only within-group differences were observed. One study found that a single session significantly reduced situational anxiety (*p* = 0.028) and personal anxiety (*p* = 0.004) [20]. Another study demonstrated a significant reduction in state anxiety following a TSB sound massage intervention (*p* < 0.05) [31]. Another study reported that sound meditation effectively reduced anxiety and tension, with first-time participants showing more pronounced improvements (*p* < 0.001) [9]. Additionally, a study involving cancer patients found that distress and autonomic anxiety-related indicators significantly decreased after six sessions of TSB sound treatment [19].

#### 3.5.2. Psychological Indicator: Depressive Symptoms

Three controlled studies showed that TSB interventions reduced depressive symptoms. One study found that both live and recorded TSB music combined with systematic desensitization significantly reduced depressive symptoms, with the live music intervention showing greater improvements (*p* < 0.001) [26]. Another study reported that a single session of TSB intervention significantly reduced depressive symptom scores on the Zung Self-Rating Depression Scale (*p* = 0.006) [29]. A randomized crossover trial observed slight improvements in the intervention group; however, the differences compared to the placebo group were not statistically significant [27]. These mixed findings resulted in moderate inconsistency of certainty assessment, leading to a downgrade in the overall certainty of evidence, despite effect sizes ranging from 0.5 to 1.8.

Two studies employed a single-group design. One study reported a significant within-group reduction in depressive symptoms, with mean scores decreasing from 0.62 to 0.42 (*p* = 0.002) [9]. In contrast, the other study did not observe a significant change in depressive symptom scores following six sessions of the intervention [19].

#### 3.5.3. Psychological Indicator: Stress

Two single-group pre-post studies on stress showed improvements in stress and related indicators following the intervention. One study reported that TSB intervention significantly reduced participants’ subjective perception of stress, with scores decreasing from 5.3 to 2.4 (*p* = 0.0005) [19]. Another study demonstrated a significant reduction in tension following a sound meditation session with TSB, with mean scores dropping from 1.26 to 0.14 (*p* < 0.001); among first-time participants, the reduction was even more pronounced, from 1.61 to 0.32 (*p* < 0.001) [9]. However, as both studies only reported within-group differences, these findings should be interpreted with caution and should not be considered definitive evidence.

#### 3.5.4. Psychological Indicator: Well-Being

One QES targeting Ukrainian refugees with PTSD reported significant improvements in well-being-related domains in the intervention group, with a 24% increase in psychological health and a 19% increase in vitality (between-group difference: *p* < 0.05) [28]. In addition, two single-group pre-post studies showed a significant within-group improvement in participants’ well-being after meditation, with SF-36 scores (*p* = 0.002) [29] or Functional Assessment of Chronic Illness Therapy–Spiritual Well-being (FACIT-SP) scores increasing from 2.85 to 3.64 (*p* < 0.001) [9]. Due to the small sample size in the single QES, the certainty of evidence was rated as low, although the estimated effect size was very large.

#### 3.5.5. Psychological Indicator: Quality of Life

Among the two controlled studies examining well-being, one reported significant improvements in vitality and psychological health in the intervention group (*p* < 0.05, with effect size 3.93) [28], while the other did not show significant improvements in quality of life [27]. A single group study also assessed quality of life but found no significant changes pre- and post-intervention [19]. Similarly, due to the substantial inconsistency between the two controlled studies, the certainty of evidence was rated as low.

#### 3.5.6. Physiological Indicator: Heart Rate Variability (HRV)

Among the four studies with a control group, three reported significant increases in RMSSD and HF (*p* < 0.05), indicating enhanced parasympathetic activity and overall improvement in HRV [25,29,30]. In contrast, one study did not find significant changes in HRV [27]. Although most of these studies demonstrated significant between-group differences, the certainty of evidence was rated as low to moderate due to the moderate risk of bias and imprecision.

Among the two studies without a control group, one showed a significant increase in HRV (from 19.7 to 22.2, *p* = 0.0041) [19]; the other reported a non-significant increase in RMSSD (from 52.4 to 55.7, *p* = 0.057) [18].

#### 3.5.7. Physiological Indicator: Heart Rate-Related Variables

Among the four studies with controls, two QES reported a significantly greater reduction in heart rate in the intervention group than in the controls (*p* < 0.05), which was associated with improvements in autonomic nervous system indicators [29,30]. The other two studies indicated that even a single-session intervention significantly reduced heart rate (*p* = 0.003; *p* = 0.009) [8,27]. A single group study also observed a significant decrease in heart rate after the intervention (*p* < 0.001) [19].

#### 3.5.8. Physiological Indicator: Brainwave Activity

One controlled study reported that the TSB group exhibited significantly reduced alpha power from T2 to T4 compared to PMR and CWL groups, with the largest difference at T3 (*p* < 0.001) [25].

Three single-group studies also observed significant within-group changes: delta increased by 135.18% (*p* = 0.001) and theta increased by 117.07% (*p* = 0.002), while alpha (85.28%, *p* = 0.005), beta (93.75%, *p* = 0.012), and gamma (81.86%, *p* < 0.001) decreased, which indicating enhanced low-frequency but suppressed high-frequency brain activity [20]. EEG analysis also showed a significant alpha power decrease in the frontal region (AF3–AF4, *p* = 0.046), while beta showed a downward trend (*p* = 0.09); delta and theta were not significantly altered, and gamma was not analyzed [19]. Another study reported significant reductions in beta-1 (*p* = 0.002), beta-2 (*p* = 0.005 and *p* < 0.001 post-intervention), and gamma (*p* = 0.001 and *p* = 0.004), with no significant changes in delta and theta waves and no detailed statistics provided for the latter [18].

### 3.6. Quality Assessment

**Figure 2:**
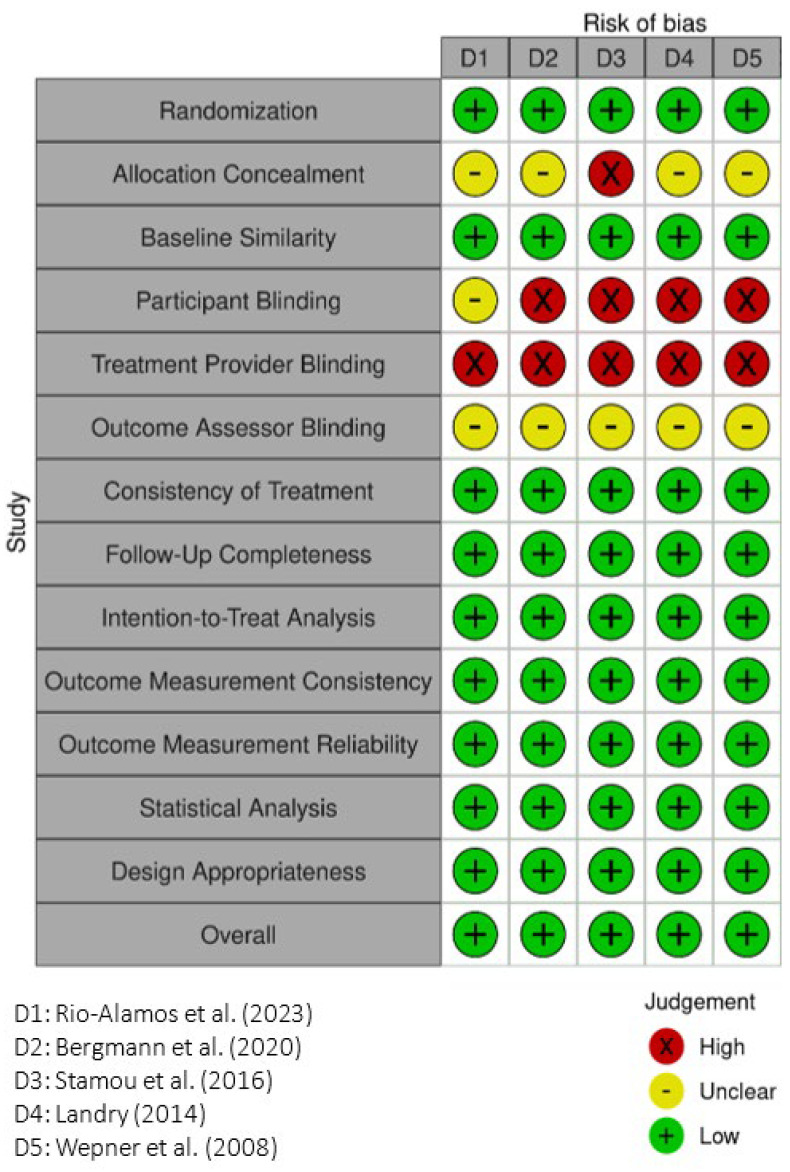
JBI critical checklist (March 2023 Edition) for randomized controlled trials. The five articles D1–D5 are referred to [25], [11], [26], [8], and [27], respectively.

As shown in Figure 2, all five RCTs were rated as having an overall low risk of bias. Most studies demonstrated adequate randomization, baseline comparability, treatment consistency, and valid statistical analysis. However, the risk of bias was frequently high or unclear regarding participant blinding, treatment provider blinding, and outcome assessor blinding. Allocation concealment was also often inadequately reported, indicating a need for improved implementation and reporting of blinding procedures.

**Figure 3:**
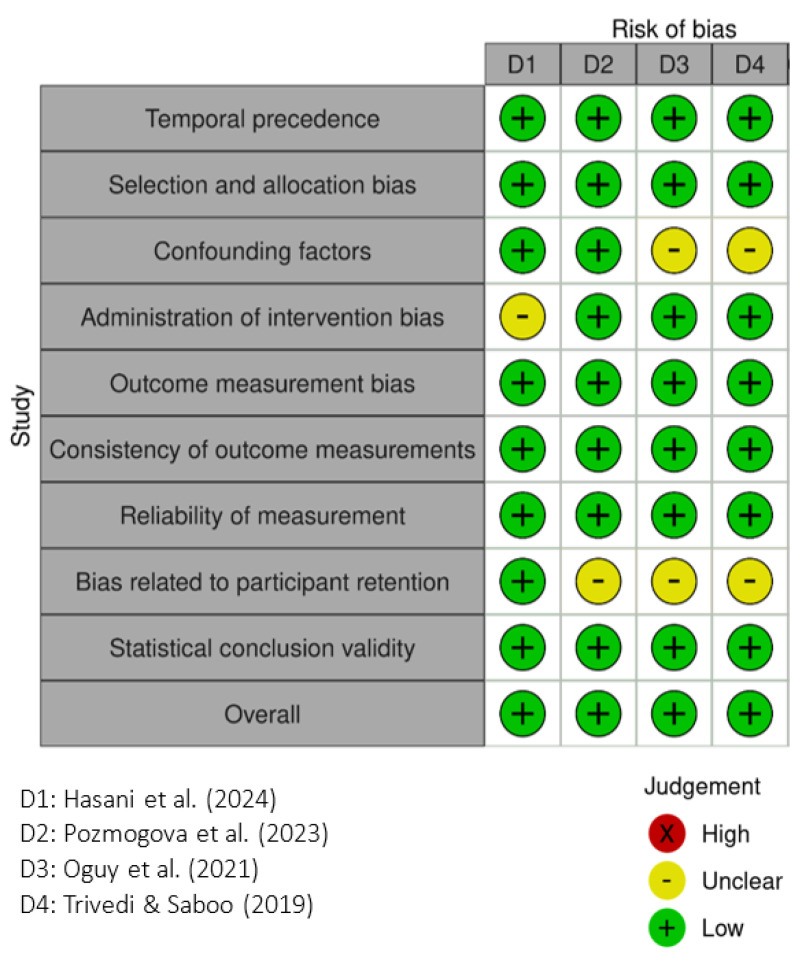
JBI critical appraisal tool (March 2024 Edition) for quasi-experimental studies—QES with controls. The four articles D1–D4 are referred to [10], [28], [29], and [30], respectively.

In Figure 3, the four QESs with comparison groups also received an overall low risk of bias rating. Although some studies lacked detailed reporting on confounding control, intervention implementation, or participant retention (resulting in several unclear ratings), other domains—including temporal sequencing, sampling strategy, measurement reliability, and statistical validity—were generally well addressed.

**Figure 4:**
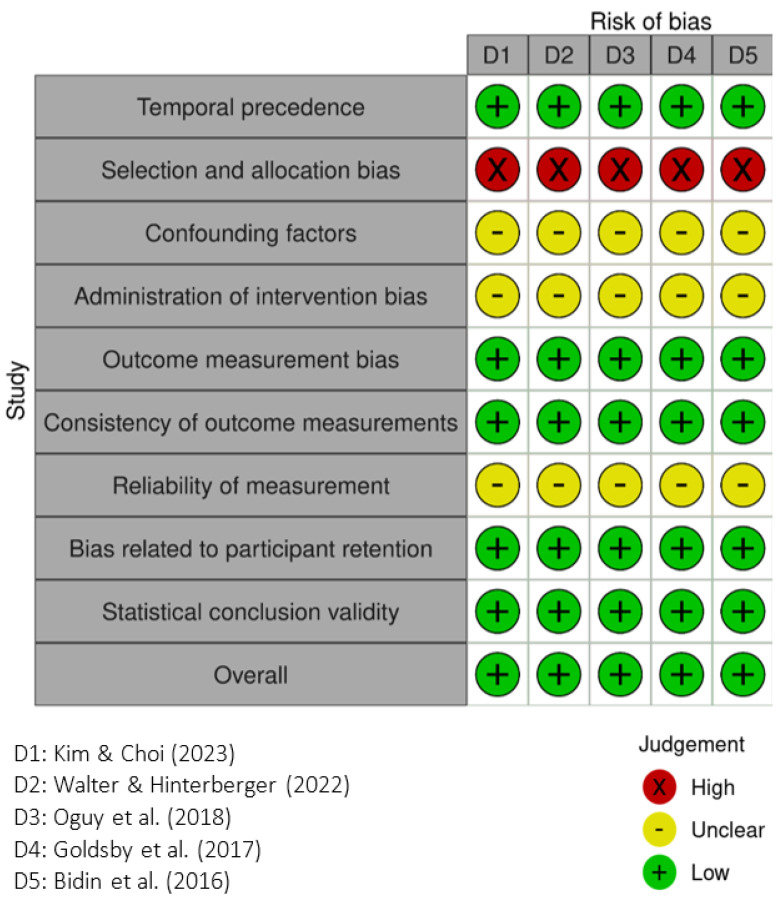
JBI critical appraisal tool (March 2024 Edition) for quasi-experimental studies—single-group pretest–posttest design. The five articles D1–D5 are referred to [20], [18], [31], [9], and [19], respectively.

In Figure 4, the five single-group pretest-posttest studies likewise received a low overall risk of bias rating. However, all studies were rated as high risk for selection bias. In addition, most studies did not sufficiently report on confounding control, intervention administration, and measurement reliability, leading to multiple unclear ratings in these domains. While statistical analysis and outcome consistency were generally rated as low risk, these findings should be interpreted with caution due to the inherent limitations of single-group designs.

Table 4 presents the Summary of Findings (SoF) along with the certainty assessment based on the GRADE framework. The certainty of evidence for the outcomes ranged from low to moderate, indicating that TSB intervention may have potential effectiveness. Given the low certainty of evidence, we excluded the results of single-group pre-post studies from the certainty assessment and evaluation of effect sizes.

## 4. Discussion

This systematic review synthesized quantitative evidence on the effects of Tibetan Singing Bowl (TSB) intervention on the psychological and physiological health of adults. It should be noted that the psychological outcomes were primarily assessed using validated self-report tools, which reflect perceived symptom severity but do not equate to clinical diagnoses. This represents a key limitation in interpreting the therapeutic implications of the findings. Nonetheless, this review identified major findings as follows.

First, the overall number of studies remains limited, with considerable methodological inconsistency in study design and intervention delivery. These studies encompassed diverse outcome measures (e.g., anxiety, HRV, brainwaves, and quality of life), but variability in intervention frequency, duration, and sample size precluded meta-analysis and limited comparability and generalizability of findings.

Second, TSB intervention appears to reduce stress, anxiety, and depressive symptoms. Most studies reported significant post-intervention decreases in anxiety and depressive symptoms, with particularly strong effects observed in high-stress populations such as surgical patients, refugees, and individuals with substance use disorders. However, given the low-to-moderate certainty of evidence, these effects should be interpreted with caution. Whether delivered in single or multiple sessions, TSB interventions helped stabilize emotions and enhance psychological well-being.

On the physiological level, many studies employed HRV, heart rate, and brainwave measures. While five studies showed increased parasympathetic indicators (e.g., HF, RMSSD, pNN50), along with reduced LF/HF ratios and heart rates, these findings were primarily derived from studies with methodological limitations and small samples, which weaken their robustness regarding neural relaxation.

Studies on brainwaves revealed specific alterations across different frequency bands. Alpha waves, associated with memory and pain modulation and protective against cognitive decline [32], were found to decrease in some studies after TSB intervention, possibly indicating reduced cognitive activity or enhanced mindfulness [18,20,25]. Beta waves, linked to alertness and cognitive processing, also generally declined, potentially reflecting reduced cognitive load and increased introspection [18,20]. Although theta waves, associated with relaxation and emotional memory, increased in some studies [20], other studies reported no significant changes [18,19], highlighting variability and limited consistency across findings despite the reported high certainty. Importantly, increased theta activity has been linked to meditative awareness and reduced cognitive arousal, suggesting a possible neurophysiological correlate of TSB-induced relaxation [33,34]

Delta waves, related to deep sleep, meditation, and physical recovery [35,36], increased under relaxed states in some studies [20], but showed no significant changes in others [18,19], again reflecting inconsistency. Nonetheless, delta enhancement has been associated with deep internalized attention and restorative processes, often observed during profound relaxation states or slow-wave meditation. Gamma waves, which reflect high-level cognitive integration and memory consolidation, decreased post-intervention in some studies—potentially indicating reduced attention and a shift toward relaxation [18,20]—while other studies did not analyze this frequency band [19], further limiting the robustness of conclusions.

Changes in EEG and HRV may help explain how the TSB method works to reduce stress. The low-frequency sounds produced by TSB may lead to neural entrainment, meaning that the brain’s electrical activity begins to align with the rhythm of the sound [37]. This process may help increase theta and delta waves, which are associated with relaxation, emotional stability, and meditative states. At the same time, reductions in alpha and beta waves, which are often associated with alertness and ongoing thinking, may reflect a calmer mental state with less cognitive activity. These brainwave changes may partly explain why many participants report feeling more relaxed after TSB sessions.

In addition, sound-induced relaxation may influence the body’s vagal tone, which reflects how well the parasympathetic nervous system is working. When vagal activity increases, people tend to feel more relaxed, and this is often shown through higher HRV and lower heart rates [38,39]. These patterns suggest that TSB may reduce stress by helping both the brain and the autonomic nervous system shift into a more relaxed state.

Finally, the TSB intervention was found to be highly acceptable, with no serious adverse effects. Participants generally reported positive experiences, including feelings of joy, safety, and connection [9,18]. The intervention is low-cost and easy to administer, making it suitable for use in non-clinical settings such as communities, schools, and stress management programs, with strong potential for wider implementation [9,25]. Moreover, its low risk, passive nature, and non-invasive characteristics make it especially suitable for individuals who cannot use medication or are unsuited for active interventions [9,25,26].

Among the studies included, two randomized controlled trials stand out for their methodological rigor [25,27]. One used a three-arm parallel design comparing TSB with progressive muscle relaxation and a passive control. It featured strong randomization and multiple physiological indicators, including HRV and EEG. However, the single-session format and use of a young, subclinical sample limit generalizability [25]. The other implemented a clinically grounded three-arm RCT involving 54 patients with chronic spinal pain, comparing TSB, a placebo (silent bowl), and no treatment. With comprehensive outcomes (e.g., SF-36, HRV, RMDQ), the study allowed a clearer assessment of TSB’s specific effects. While heart rate and pain improved, no gains were observed in functional disability or quality of life [27]. Together, these trials highlight the potential of TSB interventions while underscoring key limitations in sample representativeness, intervention duration, and clinically meaningful endpoints.

While existing studies suggest possible therapeutic benefits of TSB interventions, the evidence remains preliminary, and several limitations persist. First, there is considerable heterogeneity in research design and outcome measures, which limited meta-analysis for the evaluation of intervention effect, especially since most interventions were single-session or short-term, with only a few involving medium-term follow-ups. Second, in studies with comparison groups, the control conditions varied across studies. Meanwhile, blinding was usually not applicable. These factors could cause biased results. Therefore, interpretations of comparative effectiveness should be made with caution. Third, while some studies showed positive results, the small sample sizes limited statistical power and generalizability. Fourth, this review included only peer-reviewed, English-language, and published journal articles, excluding grey literature and non-English studies, which may have introduced selection bias. Fifth, several studies did not report detailed intervention parameters such as frequency of bowl strikes or tonal characteristics. This lack of standardization limits the ability to standardize or replicate interventions and conduct fair comparisons. Lastly, due to sparse studies, potential publication bias was not assessed. Moreover, because meta-analysis was not feasible, this could further limit the overall strength and generalizability of the evidence.

Future research should adopt standardized and replicable intervention protocols to more accurately evaluate the effectiveness of TSB interventions. This includes the use of rigorously designed controlled trials with treatment-as-usual control groups, efforts to evaluate long-term effects, recruitment of adequate sample sizes, and implementation of blinded outcome assessment where feasible. In addition, future studies should clearly define parameters such as the mode of delivery (e.g., contact-based or non-contact-based), session duration, and intervention frequency. Furthermore, the use of well-validated and widely accepted measurement tools is crucial to ensure the reliability and comparability of outcomes across studies, including objective physiological measures.

## 5. Conclusions

This review synthesized recent quantitative studies on Tibetan Singing Bowl (TSB) intervention, highlighting its potential to reduce anxiety and depressive symptoms, as well as its possible role in regulating autonomic nervous system activity and brainwaves. Given its non-invasive nature, ease of use, and high acceptability, TSB intervention may serve as a complementary therapy in both clinical and non-clinical settings. For example, it can be integrated into community mental health services as a complementary approach among individuals with mild to moderate psychological distress. Additionally, in palliative care units, TSB may provide gentle emotional relief as a non-invasive intervention for patients receiving end-of-life care. However, the current body of research is constrained by small sample sizes, heterogeneous designs, and a lack of follow-up assessment, making it difficult to draw definitive clinical recommendations at this stage. Furthermore, TSB may not be suitable for individuals with severe mental health conditions, such as those with major depressive disorder, and it should not be used as a substitute for standard medical care.

Compared to previous reviews that included only a limited number of studies or lacked methodological rigor, this review synthesizes findings from 14 original studies with quantitative data, covering diverse study designs. Risk of bias was assessed using the JBI criteria, and intervention methods, frequency, measurement tools, and psychophysiological outcomes were systematically analyzed. This synthesis addresses previous gaps in the evidence base and provides valuable reference points for future research and practical applications.

## Figures and Tables

**Table 1:** Participants and intervention programs in reviewed randomized controlled trials (RCTs).

Authors (Year) Country	Participants	Sample Size and Age: M ± SD	Intervention	Comparison	Outcome Measures	Results
Rio-Alamos et al. (2023) [25] Chile	50 adults with anxiety	TSB (*n* = 16), age: 26.2 ± 1.5. PMR (*n* = 19), age: 26.3 ± 1.0. CWL (*n* = 15), age: 25.9 ± 1.0.	One 50 min session with four Tibetan bowls, struck and rubbed to produce sound	PMR group performed muscle tensing and relaxing from feet to head for 50 min with audio guidance. CWL group sat quietly without doing anything.	SAI, HRV, EEG	TSB showed greater reductions in anxiety and EEG alpha power, and increases in HRV (HF), compared to PMR and CWL after a single session (*p* < 0.001).
Bergmann et al. (2020) [11] Austria	48 healthy adults	Median age: 31.3 Age range: 20–59	One session lying in a hammock over a 176 cm bowl, struck seven times, followed by relaxation	Silent Bowl group lay in a hammock above the bowl for 20 min without sound.	KSS, rPUI	TSB significantly reduced subjective sleepiness (*p* = 0.041), especially in women; no significant effect on objective sleepiness (*p* = 0.460).
Stamou et al. (2016) [26] Greece	24 adults in treatment for heroin addiction	IMT (*n* = 8), NIMT (*n* = 8), Control (*n* = 8). Age: 32.58 ± 5.79	Six sessions over 3 weeks: 20 min live Tibetan bowl music + 50 min guided imagery (IMT)	IMT group received singing bowl music with guided imagery twice a week for six sessions. NIMT group received recorded relaxing music with guided imagery twice a week for six sessions.	Cue-induced craving, CRS, ICT, SCL-90-R Depression subscale	IMT significantly reduced depressive symptoms (*p* < 0.001); effects generally stronger than NIMT across outcomes (permissive thoughts), though not all differences reached significance.
Landry (2014) [8] USA	51 healthy adults	crossover design (*n* = 51): HSB, Silence. Age: 50.5 ± 10.0	12 min session with bowl near the ear + 20 min directed relaxation	Silence group sat quietly for 12 min, then did 20 min of guided relaxation.	SBP, DBP, HR, PANAS	HSB significantly reduced SBP (*p* = 0.044) and HR (*p* = 0.003) compared to Silence; PANAS scores decreased significantly in both conditions (*p* < 0.001), with no between-condition differences.
Wepner et al. (2008) [27] Austria	54 adults with chronic back pain	TSB (*n* = 18), Placebo (*n* = 18), Control (*n* = 18). Age: 47.06 ± 9.33	Six sessions over 4 weeks using one selected bowl placed on painful areas and struck	Placebo group had the bowl placed on the body without sound or vibration. Control group had no intervention and continued usual care.	VAS, RMDQ, SF-36, MDBF, HR, Skin Conductance, HRV	Pain intensity and heart rate significantly decreased (*p* < 0.05); no significant effect on function (RMDQ), quality of life (SF-36), and skin conductance. Mood improved only short term (MDBF).

Abbreviations. TSB, Tibetan Singing Bowl; PMR, Progressive Muscle Relaxation; M(SD), Mean (Standard Deviation); HRV, Heart Rate Variability; SAI, State Anxiety Inventory; EEG, Electroencephalography; KSS, Karolinska Sleepiness Scale; rPUI, Relative Pupillary Unrest Index; IMT, Instrumental Music Therapy; NIMT, Non-Interactive Music Therapy; CRS, Craving Reactivity Scale; HR, Heart Rate; ICT, Impulsive and Compensatory Thoughts Questionnaire; HSB, Himalayan Singing Bowl; SBP, Systolic Blood Pressure; DBP, Diastolic Blood Pressure; PANAS, Positive and Negative Affect Schedule; VAS, Visual Analogue Scale; SF-36, Short Form Health Survey; RMDQ, Roland-Morris Disability Questionnaire; MDBF, Multidimensional Mood State Questionnaire; Skin Conductance, Skin Conductance Level.

**Table 2:** Participants and intervention programs in reviewed quasi-experimental studies with control/comparison groups.

Authors (Year) Country	Participants	Sample Size and Age: M ± SD	Intervention	Comparison	Outcome Measures	Results
Hasani et al. (2025) [10] Iran	60 adult patients awaiting coronary angiography in a hospital setting	TSB (*n* = 30), age: 54.63 ± 9.14. UC (*n* = 30), age: 57.13 ± 10.50.	Received a 10 min sound therapy using a 10 cm bowl on the palm, struck four times for 15 s each.	UC group received only routine care without sound therapy.	State Anxiety Inventory, BP, HR, RR	TSB reduced anxiety (*p* < 0.001); no significant effect on BP, HR, and RR.
Pozmogova et al. (2023) [28] Netherlands	20 adults with PTSD (Ukrainian refugees)	TSB (*n* = 10), age range:40–55. Control (*n* = 10), age range: 40–55.	Received Tibetan bowl massage with color visualization and breathing, 15–20 min per session, twice weekly for 6 weeks.	Control group received stress management only with no sound or visual input.	Short Form 36-Item Health Survey (vitality, mental health, social functioning, and emotional role functioning)	Psychological well-being improved (*p* < 0.05); control group showed no significant changes.
Oguy et al. (2021) [29] Russian Federation	81 healthy adults	Stage 1: only single-group (*n* = 19), age: 30.36 ± 13.94 Stage 2: Intervention (*n* = 31), age: 38.65 ± 9.28 Control (*n* = 31), age: 35.55 ± 4.75	Received 50 min vibroacoustic massage with a 25 cm bowl placed on the body, struck nine times per spot (1/s).	Control group received acoustic exposure only, about 2 m away with no physical contact.	Stage 1: Well-being, Activity, Mood scale, Zung, SKAS, Stage 2: HRV, hemodynamics	Well-being, depressive symptoms, and anxiety improved (*p* < 0.01); mood nonsignificant (*p* = 0.244).
Trivedi & Saboo (2019) [30] India	33 adults with sleep or stress-related concerns	HSB (*n* = 16), mean age: 28 SS (*n* = 17), mean age: 25	HSB group received a 20 min session with singing bowls and Tingsha	SS group rested in a supine position for 20 min without sound stimulation	Stress Index, HR, RMSSD	Stress Index and HR decreased, RMSSD increased (*p* < 0.05); control group showed no significant changes.

Abbreviations. TSB, Tibetan Singing Bowl; M(SD), Mean (Standard Deviation); UC, Usual Care; BP, Blood Pressure; HR, Heart Rate; RR, Respiratory Rate; HSB, Himalayan Singing Bowl; SS, Supine Silence; HRV, Heart Rate Variability; RMSSD, Root Mean Square of the Successive Differences; Zung, Zung Self-Rating Depression Scale; SKAS, Spielberger-Khanin Anxiety Scale; SF-36, Short Form 36-Item Health Survey.

**Table 3:** Characteristics of participants and intervention programs in studies with a single interventional group.

Authors (Year) Country	Participants	Sample Size and Age: M ± SD	Intervention	Outcomes	Results
Kim & Choi (2023) [20] Republic of Korea	General adults	*n* = 17, age: 25.2 ± 3.5	5 min non-contact bowl striking (260 mm × 115 mm bowl, struck six times at 50 sec intervals)	EEG (Delta, Theta, Alpha, Beta, Gamma)	Delta and theta waves increased (135.2% and 117.1%), and 6.68 Hz beat rose by 251.98%. Alpha, beta, and gamma waves decreased (14.7%, 6.3%, and 18.1%). Effects remained after the sound stopped.
Walter & Hinterberger (2022) [18] Germany	General adults	*n* = 34, age: 36.0 ± 13.4	20 min on-body singing bowl massage following the Peter Hess^®^ method	EEG, HRV, HR, RR, TAS, CSP-14	EEG global power decreased (*p* < 0.001), especially in Beta 2 (*p* = 0.002) and Gamma (*p* = 0.004); HR significantly decreased (*p* < 0.001); subjective well-being improved per CSP-14; HRV changes were not significant (e.g., RMSSD *p* = 0.057)
Oguy et al. (2018) [31] Russia	Adults with stress-related symptoms	*n* = 33, age: 35.0 ± 6.3	2–4 vibroacoustic massage sessions (30–70 min) using “giving” and “receiving” bowl striking techniques	ECG, HR, HRV, ZSAS, STAI	Anxiety significantly decreased after the intervention, with ZSAS dropping from 48.2 to 35.6 and STAI-SA from 39.8 to 26.3 (*p* < 0.05); however, symptoms partially returned after 24 h with no significant difference (*p* > 0.05).
Goldsby et al. (2017) [9] USA	General adults	*n* = 62, age: 49.7 ± 13.0	60 min sound meditation using ~95% Jambati bowls and other instruments; participants lay down surrounded by bowls.	POMS-SF, HADS, FACIT-SP, pain rating	Significant reductions in tension, anger, confusion, fatigue, anxiety, and depressive symptoms (*p* < 0.001); increased spiritual well-being (*p* < 0.01)
Bidin et al. (2016) [19] Italy	Adult patients with cancer	*n* = 12, (age not reported)	Six 60 min Bagno Armonico sessions over 3 months with on-body application of bowls, gongs, bells, and chimes; nonverbal; assessed for subjective and physiological outcomes	SF-36, PDI, HADS, DT, SCL, HRV, FACIT-F, EEG	TSB reduced distress (*p* = 0.0005), tonic and phasic SCL (*p* = 0.0091 and 0.0064), resting heart rate (*p* = 0.0001); increased HRV and oxygenation (*p* = 0.0041 and 0.0003). EEG showed decreased alpha and beta (*p* = 0.046 and 0.090), increased coherence (*p* = 0.084). Others not significant.

Abbreviations. M(SD), Mean (Standard Deviation); EEG, Electroencephalography; HRV, Heart Rate Variability; HR, Heart Rate; RR, Respiratory Rate; TAS, Toronto Alexithymia Scale; CSP-14, Core Seven Emotions and Three Somatic Sensations Scale (14-item version); ECG, Electrocardiography; ZSAS, Zung Self-rating Anxiety Scale; STAI, State-Trait Anxiety Inventory; POMS-SF, Profile of Mood States–Short Form; HADS, Hospital Anxiety and Depression Scale; FACIT-SP, Functional Assessment of Chronic Illness Therapy–Spiritual Well-being; SF-36, Short Form-36 Health Survey; PDI, Pain Distress Index; DT, Distress Thermometer; SCL, Skin Conductance Level; FACIT-F, Functional Assessment of Chronic Illness Therapy-Fatigue.

**Table 4:** GRADE-based evidence assessment of TSB interventions.

Outcome	No. of Studies	Risk of Bias	Certainty of Assessment ^a^ Inconsistency Indirectness Imprecision			No. of Participants ^a^	Effect Size ^b^	Certainty
Anxiety	1 RCT 1 QES 4 single-group	Low	No serious	Not serious	No serious	110	1.51 and 2.18	Moderate
Depressive symptoms	2 RCT 1 QES 2 single-group	Moderate	Moderate	No serious	No serious	97	Between 0.5 and 1.8	Low to Moderate
Well-Being	1 QES 1 single-group	Moderate	NA	No serious	Serious	20	2.1	Low
Quality of Life	1 RCT 1 QES 1 single-group	Moderate	Serious	Low	Serious	74	0.09 and 3.93	Low
Heart Rate Variability: RMSSD	2 RCT 2 QES 2 single-group	Moderate	No serious	Low	Moderate	181	Between 0.6 and 0.8	Low to Moderate
Heart Rate	2 RCT 2 QES 2 single-group	Moderate	No serious	Low	Moderate	182	Between −0.5 and 0.8	Low
Brainwave Activity	1 RCT 3 single-group	Low	No serious	No serious	No serious	50	1.3	Low to Moderate

Abbreviations. RCT, Randomized Controlled Trials; QES, Quasi-Experimental Studies (with controls). ^a^ Considering there was no control group in the single-group study, only RCT and QES were assessed. ^b^ Due to meta-analysis not being feasible, the effect size presented here was estimated for each study of RCT and QES when available.

## Data Availability

Data sharing is not applicable. No new data were created or analyzed in this study.

## Supplementary Materials

The following supporting information can be downloaded at: https://www.mdpi.com/article/10.3390/healthcare13162002/s1, Table S1: Search strings for various databases; Table S2: Details of the excluded articles.
