# Supplementary material for: Effects of Tibetan Singing Bowl Intervention on Psychological and Physiological Health in Adults: A Systematic Review
Source: Healthcare (Basel). 2025 Aug 14;13(16):2002. doi: 10.3390/healthcare13162002 (PMC12385955; doi:10.3390/healthcare13162002)
Supplement: Supplementary file 1 [file healthcare-13-02002-s001.zip › healthcare-3713201-supplementary.pdf]

Supplementary Table S1. Search strings for various databases

**Search terms for MEDLINE (via Ovid)**
("Singing Bowl" OR "Tibetan Bowl").ti,ab.<br>Filters: Peer-Reviewed Journals, English Language, Publication Date: 2000/01/01–2025/03/31
**Search terms for PubMed**
("Singing Bowl"[Title/Abstract] OR "Tibetan Bowl"[Title/Abstract])<br>AND ("2000/01/01"[Date - Publication] : "2025/03/31"[Date - Publication])<br>Filters: English, Journal Article, Humans
**Search terms for Scopus**
TITLE-ABS-KEY("Singing Bowl" OR "Tibetan Bowl")<br>AND PUBYEAR = 2000 TO 2025<br>AND (LIMIT-TO(DOCTYPE, "ar"))<br>AND (LIMIT-TO(LANGUAGE, "English"))
**Search terms for EMBASE (via Elsevier)**
('singing bowl':ti,ab,kw OR 'tibetan bowl':ti,ab,kw)<br>AND [2000-2025]/py<br>AND [english]/lim<br>AND [article]/lim
**Search terms for CINAHL (via EBSCOhost)**
TX ("Singing Bowl" OR "Tibetan Bowl")<br>AND (Publication Date: 20000101–20250331)<br>Limiters: Peer Reviewed; English Language; Journal Articles Only
**Search terms for Cochrane Library**
("Singing Bowl" OR "Tibetan Bowl"):ti,ab,kw<br>Publication Date: 2000/01/01 to 2025/03/31<br>Filters applied: Trials and Reviews; English

Supplementary Table S2. Details of the excluded articles

First author	Year	Title	Reasons for exclusion
R. Seetharaman	2024	Exploring the healing power of singing bowls: An overview of key findings and potential benefits	Review article
J. Stanhope	2020	The human health effects of singing bowls: A systematic review	Review article
M. Burtne	2002	REAL-TIME SPATIAL PROCESSING AND TRANSFORMATIONS OF A SINGING BOWL	Not relevant to TSB intervention
R. Aarts	2014	An electro-acoustic implementation of Tibetan bowls: Acoustics and perception	Not relevant to TSB intervention
S. Nasrat	2024	Multiscaled crucial events complexity analysis of heart rate signals during Tibetan singing bowls meditation	Not relevant to TSB intervention
C. R. Rashmi	2024	Evaluating Deep Learning with different feature scaling techniques for EEG-based Music Entrainment Brain Computer Interface	Not relevant to TSB intervention
R. Prasad	2024	Participants tend to Synchronize with the Tibetan Singing Bowl	Not relevant to TSB intervention
G. Essl	2002	Banded Waveguides on Circular Topologies and of Beating Modes: Tibetan Singing Bowls and Glass Harmonicas	Not relevant to TSB intervention
A. Gołas	2016	Digital synthesis of sound generated by Tibetan bowls and bells	Not relevant to TSB intervention
O. Inácio	2006	The dynamics of Tibetan singing bowls	Not relevant to TSB intervention
B. S. Limkar	2023	Structural dynamic analysis of a musical instrument: Tibetan singing bowl	Not relevant to TSB intervention
B. T. Wang	2018	Vibration modes and sound characteristic analysis for different sizes of singing bowls	Not relevant to TSB intervention
O. T. Wee	2023	Operational Modal Analysis of Tibetan Singing Bowl	Not relevant to TSB intervention

C. Río-Alamos	2022	Measuring the anxiety-reducing effects of Tibetan singing bowls	A registered protocol
S. Shahid	2022	Efficacy of Singing Bowls in Childhood Asthma—A Pilot and Feasibility Study	Involved a pediatric population
